# Enhancing Stability in Next‐Generation Photoelectrodes Such as Organic Semiconductor and Halide Perovskite‐Based Photoelectrodes for Various Applications: Recent Advanced Passivation and Encapsulation Methods

**DOI:** 10.1002/EXP.20240020

**Published:** 2026-01-29

**Authors:** Yejoon Kim, Enok Lee, Sanghan Lee

**Affiliations:** ^1^ Department of Materials Science and Engineering Gwangju Institute of Science and Technology Gwangju Republic of Korea; ^2^ Research Institute for Solar and Sustainable Energies Gwangju Institute of Science and Technology Gwangju Republic of Korea

**Keywords:** bias‐free system, encapsulation, halide perovskite, organic semiconductor, passivation, photoelectrochemical

## Abstract

This review explores advancements in passivation and encapsulation methods for organic semiconductor (OS) and halide perovskite (HP)‐based photoelectrodes, aimed at enhancing stability in photoelectrochemical (PEC) systems. The integration of OS and HP materials in PEC devices offers high photovoltage and impressive current densities, promising efficient solar‐to‐fuel conversion and pollutant upcycling. However, these materials face significant intrinsic and extrinsic degradation under PEC conditions. We discuss various passivation strategies to mitigate photochemical reactions and defect‐related degradation, as well as advanced encapsulation techniques to prevent electrolyte‐induced corrosion. The review highlights the latest research on enhancing the operational stability and performance of OS and HP‐based photoelectrodes, emphasizing their potential in sustainable energy applications and environmental remediation. Future research directions include optimizing material design, exploring new passivation and encapsulation technologies, and scaling up for practical applications. This comprehensive review underscores the critical role of material innovation in advancing PEC technology for a sustainable future.

## Graphical abstract

This review explores advancements in passivation and encapsulation methods to enhance the stability of organic semiconductor and halide perovskite‐based photoelectrodes in photoelectrochemical systems. It highlights the potential of these materials for efficient solar energy conversion and environmental applications while emphasizing the need for further research in optimizing material design and developing new technologies for practical use.

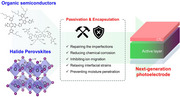

## 1 Introduction

As global energy demand continues to rise, the pursuit of sustainable energy solutions has become increasingly urgent, leading to active exploration of innovative technologies that leverage solar power for efficient energy conversion [1, 2, 3]. Among these technologies, photoelectrochemical (PEC) systems, which integrate photovoltaic (PV) and electrochemical (EC) cells into a single device using photoelectrodes, show significant potential for directly converting solar energy into storable fuels without intermediate steps [4, 5, 6]. Consequently, the photoelectric and electrochemical properties of the photoelectrodes directly impact the overall system performance. Notably, the photoelectric properties, represented by the open‐circuit voltage and short‐circuit current density of the photoelectrodes, are the primary factors in evaluating the performance of PEC systems. However, conventional PEC systems based on silicon [7, 8], III–V semiconductors [9, 10], and metal oxide photoelectrodes [11, 12, 13] have demonstrated unsatisfactory photoelectric properties, often relying on external bias to drive redox reactions. This dependency introduces issues related to energy efficiency, system complexity, and cost‐effectiveness. To address these challenges and create an ideal PEC system that operates without external voltage, efforts have been made to convert next‐generation solar cells with superior photoelectric properties into photoelectrodes [14]. Specifically, organic semiconductor (OS) and halide perovskite (HP)‐based photoelectrodes have garnered significant attention due to their potential for large‐scale commercialization. Both materials exhibit excellent light absorption across a broad spectrum and can be tuned to achieve desirable properties for PEC systems [15, 16, 17]. Additionally, solution printing processes based on organic solvents make them suitable for low‐cost, large‐area device fabrication [18, 19]. OS and HP‐based photoelectrodes have demonstrated remarkable photoelectric properties, including open‐circuit voltages exceeding 1.0 V and current densities in the tens of mA cm^−2^, along with unparalleled solar conversion efficiencies compared to other single light‐absorbing layer materials [20, 21, 22, 23, 24].

However, early OS and HP‐based photoelectrodes exhibited extremely poor operational stability, completely failing within hours, compared to the hundreds of hours of operational time demonstrated by conventional photoelectrodes [25, 26, 27, 28]. This stability issue arises from the harsh operating conditions of PEC systems, which differ significantly from those of PV systems. In PEC systems, the external circuit between the two electrodes is composed of an electrolyte, which relies on ion conduction rather than the rapid electron transfer that occurs in traditional metal wires [29]. For ion conduction to occur, electrochemical reactions must take place, introducing significant resistance to the overall circuit and causing charge accumulation within the device. Defects in the materials comprising the photoelectrodes serve as recombination sites for the accumulated charges, initiating degradation [30, 31]. Consequently, the stability of OS and HP‐based photoelectrodes is much lower than that of PV devices with the same structure, with degradation occurring more frequently and extensively. Therefore, the development of highly stable OS and HP PV devices is essential before converting them into photoelectrodes. This requires a deep understanding of the materials and the introduction of meticulous passivation techniques.

**FIGURE 1:**
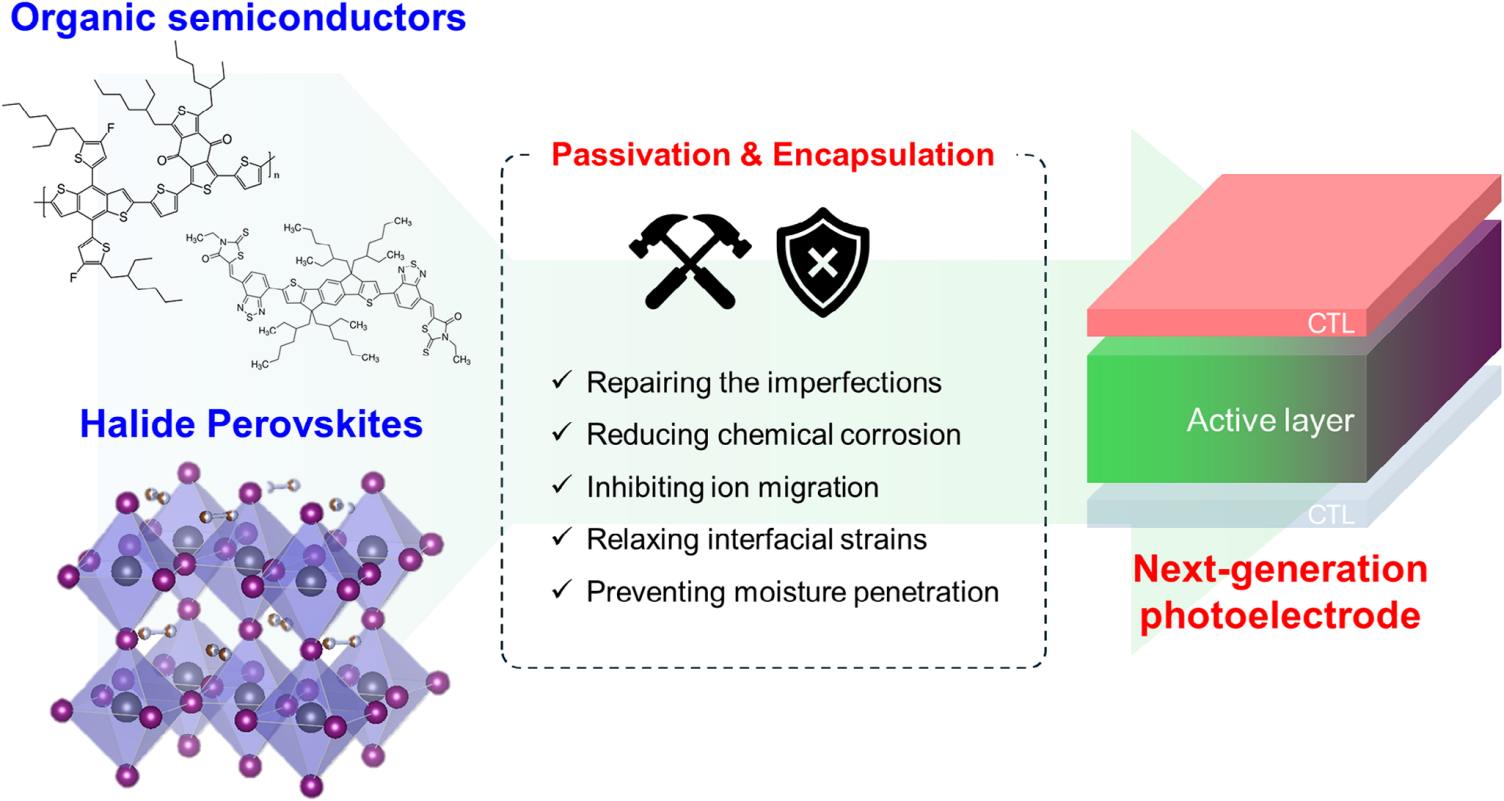
Strategies for OS and HP passivation and encapsulation to fabricate next‐generation photoelectrodes.

Moreover, photoelectrodes operating in electrolytes face inevitable corrosion or decomposition in aqueous electrolytes, to which OS and HP‐based photoelectrodes are particularly vulnerable. While conventional photoelectrodes also experience electrochemical corrosion in aqueous electrolytes, they tend to remain relatively stable in the absence of external voltage due to their strong covalent bonds. In contrast, OS and HP‐based photoelectrodes, typically fabricated through solution coating processes with organic solvents, cannot withstand such conditions and decompose in the electrolyte, leading to immediate device failure [32, 33]. Therefore, when converting OS and HP PV devices into photoelectrodes, it is very important to apply encapsulation methods that can prevent the infiltration of aqueous electrolytes. These encapsulation methods should be designed to minimize internal resistance and performance degradation (Figure 1).

In this review, we explore the structures of solar cells and photoelectrodes with single OS and HP light‐absorbing layers, investigate degradation mechanisms that may occur in each structure, and discuss suitable passivation methods to achieve high operational stability in electrolyte environments. Additionally, we summarize various encapsulation methods to block the penetration of aqueous electrolytes, assessing the advantages and disadvantages of each approach. Finally, we highlight various PEC systems, such as water splitting and CO_2_ reduction, utilizing OS and HP‐based photoelectrodes. We expect that focusing on the design and development of OS and HP‐based photoelectrodes will lead to significant advancements in the environmentally friendly PEC field.

## 2 Structure of OS and HP‐Based Photoelectrodes

The basic structure of OS and HP‐based photoelectrodes is the same as that of solar cells. It follows a sandwich structure where the charge transport layers and electrodes are sequentially positioned around the light‐absorbing layer [34, 35]. This structure is carefully crafted considering the overall energy band diagram, promoting effective separation of electrons and holes, and their conversion into current and power [36, 37]. In this chapter, to ensure a smooth understanding of the overall review and provide fundamental insights into device design, we will explore the principles and representative materials used in the structure of OS and HP‐based devices.

### 2.1 Light‐Absorbing Layer

The light‐absorbing layer, also known as the ‘active layer,’ is the most distinctive structure of OS and HP‐based photoelectrodes. It absorbs sunlight to generate charge carriers (electrons and holes), which then move along the electric field to form a current [38, 39]. The active layer plays a crucial role in both types of photoelectrodes, absorbing sunlight and generating electrical energy, each comprising unique materials and structures. Commonly used materials are summarized in Table 1. Additionally, the efficiency of the active layer is a critical factor determining the overall performance of solar cells [40, 41].

**TABLE 1:** Summarization of the OS and HP‐based active layer materials.

Organic semiconductor‐based active layer	
Type	Materials
Polymer donors	P3HT^a^, [42] PTB7‐Th^b^ [43], PCDTBT^c^ [44], PBDB‐T‐2F^d^ [45], PffBT4T^e^ [46], PBDB‐T^f^ [47], etc.
Small molecule donors	DPP‐DTT^g^ [48], BDT^h^ [49], IT‐4F^i^ [50], SM1^j^ [51], SM2^k^ [51], etc.
Fullerene acceptors	PCBM^l^ [52], PC_71_BM^m^ [53], other C_60_‐derivatives [54], etc.
Non‐fullerene acceptors	ITIC^n^ [47], BTP‐4F^o^ [55], IDTBR^p^ [42], IDIC^q^ [56], ITIC‐M^r^ [57], IEICO‐4F^s^ [58], FBR^t^ [59], etc.

Halide perovskite active layer	
Type	Materials
Halide perovskites	MAPbI_3_‐Br_3_‐Cl_3_ [60, 61, 62], FAPbI_3_‐Br_3_‐Cl_3_ [63, 64, 65], CsPbI_3_‐Br_3_‐Cl_3_ [66, 67, 68], etc.

^a^ (Poly([2,6′‐4,8‐di(5‐ethylhexylthienyl)benzo[1,2‐b;3,3‐b]dithiophene][3‐fluoro‐2[(2‐ethylhexyl)carbonyl]thieno[3,4‐b]thiophenediyl])).

^b^ (Poly(3‐hexylthiophene)).

^c^ (Poly[*N*‐9′‐heptadecanyl‐2,7‐carbazole‐*alt*‐5,5‐(4′,7′‐di‐2‐thienyl‐2′,1′,3′‐benzothiadiazole)], Poly[[9‐(1‐octylnonyl)‐9H‐carbazole‐2,7‐diyl]‐2,5‐thiophenediyl‐2,1,3‐benzothiadiazole‐4,7‐diyl‐2,5‐thiophenediyl]).

^d^ (Poly[[4,8‐bis[5‐(2‐ethylhexyl)‐4‐fluoro‐2‐thienyl]benzo[1,2‐*b*:4,5‐*b*′]dithiophene‐2,6‐diyl]‐2,5‐thiophenediyl[5,7‐bis(2‐ethylhexyl)‐4,8‐dioxo‐4*H*,8*H*‐benzo[1,2‐*c*:4,5‐*c*′]dithiophene‐1,3‐diyl]‐2,5‐thiophenediyl]).

^e^ (Poly[(5,6‐difluoro‐2,1,3‐benzothiadiazole‐4,7‐diyl)).

^f^ (Poly[[4,8‐bis[5‐(2‐ethylhexyl)‐2‐thienyl]benzo[1,2‐*b*:4,5‐*b*′]dithiophene‐2,6‐diyl]‐2,5‐thiophenediyl[5,7‐bis(2‐ethylhexyl)‐4,8‐dioxo‐4*H*,8*H*‐benzo[1,2‐*c*:4,5‐*c*′]dithiophene‐1,3‐diyl]] polymer).

^g^ (Poly[2,5‐(2‐octyldodecyl)‐3,6‐diketopyrrolopyrrole‐alt‐5,5‐(2,5‐di(thien‐2‐yl)thieno [3,2‐b]thiophene)]).

^h^ (Benzo[1,2‐b:4,5‐b′]dithiophene).

^i^ (3,9‐bis(2‐methylene‐((3‐(1,1‐dicyanomethylene)‐6,7‐difluoro)‐indanone))‐5,5,11,11‐tetrakis(4‐hexylphenyl)‐dithieno[2,3‐d:2’,3’‐d’]‐s‐indaceno[1,2‐b:5,6‐b’]dithiophene).

^j^ (small molecule 1).

^k^ (small molecule 2).

^l^ (Phenyl‐C61‐butyric acid methyl ester).

^m^ (Phenyl‐C71‐butyric acid methyl ester).

^n^ (3,9‐bis(2‐methylene‐(3‐(1,1‐dicyanomethylene)‐indanone))‐5,5,11,11‐tetrakis(4‐hexylphenyl)‐dithieno[2,3‐d:2′,3′‐d′]‐s‐indaceno[1,2‐b:5,6‐b′]dithiophene).

^o^ (2,2′‐[[12,13‐Bis(2‐ethylhexyl)‐12,13‐dihydro‐3,9‐diundecylbisthieno[2′′,3′′:4′,5′]thieno[2′,3′:4,5]pyrrolo[3,2‐e:2′,3′‐g][2,1,3]benzothiadiazole‐2,10‐diyl]bis[methylidyne(5,6‐difluoro‐3‐oxo‐1H‐indene‐2,1(3H)‐diylidene)]]bis[propanedinitrile]).

^p^ ((5Z,5′Z)‐5,5′‐((7,7′‐(4,4,9,9‐tetraoctyl‐4,9‐dihydro‐s‐indaceno[1,2‐b:5,6‐b′]dithiophene‐2,7‐diyl)bis(benzo[c][1,2,5]thiadiazole‐7,4‐diyl))bis(methanylylidene))bis(3‐ethyl‐2‐thioxothiazolidin‐4‐one)).

^q^ (2,2'‐((2Z,2'Z)‐((4,4,9,9‐tetrahexyl‐4,9‐dihydro‐s‐indaceno[1,2‐b:5,6‐b']dithiophene‐2,7‐diyl)bis(methanylylidene))bis(3‐oxo‐2,3‐dihydro‐1H‐indene‐2,1‐diylidene))dimalononitrile).

^r^ (3,9‐bis(2‐methylene‐((3‐(1,1‐dicyanomethylene)‐6/7‐methyl)‐indanone))‐5,5,11,11‐tetrakis(4‐hexylphenyl)‐dithieno[2,3‐d:2’,3’‐d’]‐s‐indaceno[1,2‐b:5,6‐b’]dithiophene).

^s^ (2,2'‐((2Z,2'Z)‐(((4,4,9,9‐tetrakis(4‐hexylphenyl)‐4,9‐dihydro‐sindaceno[1,2‐b:5,6‐b′]dithiophene‐2,7‐diyl)bis(4‐((2‐ethylhexyl)oxy)thiophene‐5,2‐diyl))bis(methanylylidene))bis(5,6‐difluoro‐3‐oxo‐2,3‐dihydro‐1H‐indene‐2,1‐diylidene))dimalononitrile).

^t^ (5,5′‐[(9,9‐Dioctyl‐9*H*‐fluorene‐2,7‐diyl)bis(2,1,3‐benzothiadiazole‐7,4‐diylmethylidyne)]bis[3‐ethyl‐2‐thioxo‐4‐thiazolidinone]).

In OS‐based active layers, donor and acceptor materials are typically composed of polymers and fullerene derivatives [69, 70]. The donor donates electrons, while the acceptor receives electrons. The bulk‐heterojunction structure, where donor and acceptor materials are mixed on a nanometer scale, enhances light absorption and charge separation efficiency [69, 71]. When sunlight is absorbed by the donor material, excitons (electron–hole pairs) are generated. These excitons are separated into electrons and holes at the donor–‐acceptor interface [72]. The separated electrons and holes then move to the electrodes through the acceptor and donor materials, respectively, forming a current through the external circuit.

The active layer in HP‐based photoelectrodes consists of materials with a halide perovskite structure of ABX_3_ [73]. Here, A‐site represents an organic/inorganic cation (e.g., methylammonium (MA), formamidinium (FA), Cs^+^, Rb^+^), B‐site represents a metal cation (e.g., Pb^2+^, Sn^2+^), and X‐site represents a halide anion (e.g., I^−^, Br^−^, Cl^−^). The formation of the ABX_3_ crystal structure is determined by the tolerance factor, *t*, which is defined by the formula below;

$$t=\left(rA+rX\right)/[\sqrt{2}\left(rB+rX\right)]$$

where *r*A and *r*B are the ionic radii of the A‐site and B‐site cations, respectively, and *r*X is the ionic radius of the X‐site anion [74]. It is generally reported that perovskites form in the range 0.8 ≤ *t* ≤ 1. Uniquely, the bandgap of the HP‐based active layers can be tuned freely by changing or mixing the halide X‐site [75].

### 2.2 Charge Transport Layer

The charge transport layer (CTL) is located above and below the active layer, efficiently extracting charge carriers (electrons and holes) and transporting them to the respective electrodes. This is essential for generating current and enhancing power conversion efficiency. CTL is divided into the electron transport layer and the hole transport layer, each promoting the movement of electrons and holes, respectively.

- Electron transport layer (ETL): ETL collects electrons generated in the active layer and transfers them to the cathode. ETL must have high electron mobility and suitable energy levels, minimizing electron recombination to enhance electron transport efficiency [76, 77, 78]. It is typically composed of n‐type semiconductor materials.
- Hole transport layer (HTL): HTL collects holes generated in the active layer and transfers them to the anode. HTL must have high hole mobility and suitable energy levels, minimizing hole recombination to enhance hole transport efficiency [79]. It is typically composed of p‐type semiconductor materials.

The CTL provides pathways for the movement of electrons and holes, minimizing recombination and optimizing charge transport to the electrodes, thereby increasing the efficiency of solar cells. Additionally, the CTL adjusts the energy barriers between the active layer and the electrodes to facilitate the extraction of electrons and holes. The materials used for the CTL should be appropriately selected based on the energy band structure of the active layer [78, 79, 80]. Popular materials for ETL and HTL are summarized in Table 2.

**TABLE 2:** Summarization of the charge transport layer materials.

Type	Materials
Electron transport layer	TiO_2_ [81], ZnO [82], SnO_2_ [83], PCBM^a^ [84], Al_2_O_3_ [85], CdS [86], ZnS [87], LiF [88], etc.
Hole transport layer	PEDOT:PSS^b^ [89], MoO_3_ [90], Spiro‐OMeTAD^c^ [91], NiO [92], CuI [93], WS_2_ [94], etc.

^a^ (Phenyl‐C61‐butyric acid methyl ester).

^b^ (Poly(2,3‐dihydrothieno‐1,4‐dioxin)‐poly(styrenesulfonate)).

^c^ (2,2′,7,7′‐tetrakis[*N*,*N*‐di(4‐methoxyphenyl)amino]‐9,9′‐spirobifluorene.

### 2.3 Electrodes

Electrodes, anodes and cathodes, play a crucial role in collecting electrons and holes and transferring them to the external circuit. To allow the active layer to absorb light, one electrode must be a transparent electrode with high transmittance [95]. Typically, transparent conductive materials with high optical transparency and low resistance are used. Representative materials include indium tin oxide (ITO) and fluorine‐doped tin oxide (FTO).

For the anode, materials with a high work function are necessary to facilitate hole collection. These materials align well with the energy levels of the HTL, aiding in the effective extraction of holes. Examples include gold (Au) [96], platinum (Pt) [97], and nickel (Ni) [97]. The cathode, which collects electrons and transfers them to the external circuit, utilizes metals with a low work function to optimize electron collection. These materials match the energy levels of the ETL, promoting efficient electron extraction and transport. Common examples are aluminum (Al) [98], silver (Ag) [99], and calcium (Ca) [100]. High work function materials facilitate hole extraction in the HTL, while low work function materials ease electron extraction in the ETL, minimizing energy barriers, and optimizing charge transport pathways.

## 3 Intrinsic Degradation Mechanisms and Passivation Methods

Intrinsic degradation phenomena in OS and HP‐based photoelectrodes can be broadly classified into three categories based on their location. One occurs within the active layer, while the other two occur at the interfaces between the active layer and the HTL and ETL. Firstly, degradation within the active layer is primarily due to photochemical reactions induced by light [101, 102]. This includes the direct decomposition reactions of the light‐absorbing material caused by UV light, and structural changes within the active layer resulting from the movement of photoexcited electrons and holes, as well as ion migration induced by light irradiation [103, 104, 105]. Secondly, degradation at the interfaces is mainly due to abnormal material and charge transport phenomena occurring at the material interfaces [106]. This includes non‐radiative recombination of electrons and holes and corrosion reactions arising from abnormal or incidental reactions at the interfaces [107, 108, 109]. Overall, various degradation phenomena can be understood as abnormal reactions occurring near imperfections within the material and at the interfaces [110, 111, 112]. Therefore, to address these issues, passivation is required to repair and complement the imperfections in the materials used in the active layer, HTL, and ETL. In this chapter, we will delve into the mechanisms of degradation phenomena occurring at each location and discuss various passivation methods to enhance the operational stability of OS and HP devices.

### 3.1 Active Layer

#### 3.1.1 Organic Semiconductors

In OS‐based solar cells and photoelectrodes, a peculiar phenomenon is observed where the device rapidly degrades during the initial few hundred hours, followed by relatively stable operation for several thousand hours. This initial phase of degradation is referred to as “burn‐in” and effectively mitigating burn‐in is crucial for determining the overall efficiency of the device. Several mechanisms have been proposed to explain this anomaly, including the influence of impurities [113, 114], the crystallinity of organic semiconductor materials [115], and the indirect effects of polymer chain ends [116]. However, the most prominent explanation attributes this phenomenon to the use of fullerene acceptors. It has been reported that fullerenes form dimers through photochemical reactions, creating exciton traps that hinder efficient separation and collection [117, 118].

**FIGURE 2:**
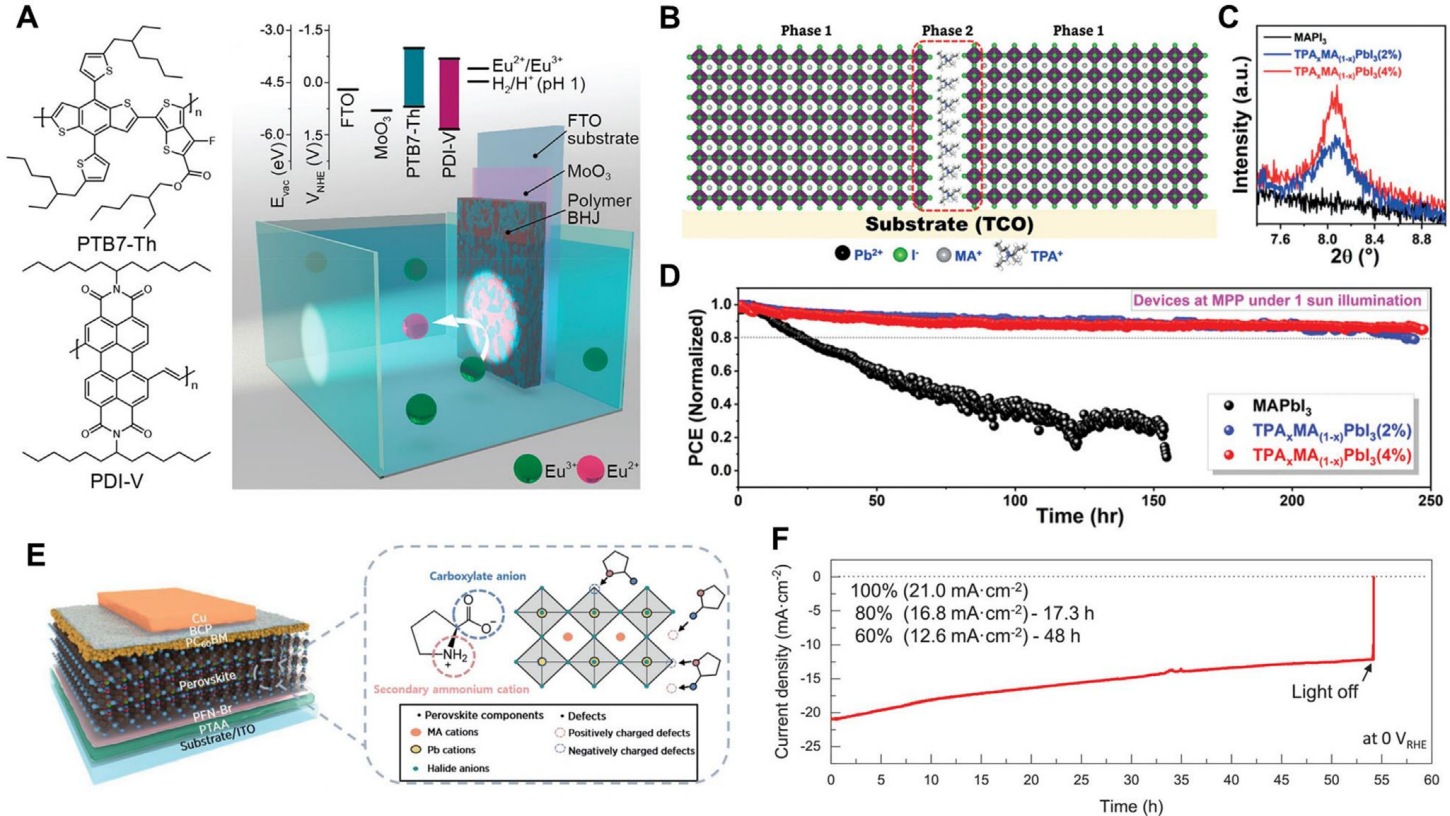
(A) Chemical structures of PTB7‐Th polymer donor and PDI‐V NFAs, and schematic image of OS‐based photocathode. Reproduced with permission [121]. Copyright 2020, American Chemical Society. (B–D) Schematic of perovskite passivation by TPA^+^ ion injection, XRD spectra of bare MAPbI_3_ film and TPA^+^ ion treated MAPbI_3_ films, and operational stability of unencapsulated devices based on MAPbI_3_, and TPA^+^ ion treated MAPbI_3_. Reproduced with permission [122]. Copyright 2020, Wiley‐VCH. (E, F) Schematic image of HP‐based photocathode passivated by the Pro zwitterion, and the PEC operational stability of the Pro zwitterion treated HP‐based photocathode. Reproduced with permission [123]. Copyright 2021, Wiley‐VCH.

Consequently, the use of higher adduct fullerene acceptors that do not form dimers, or non‐fullerene acceptors (NFAs), can effectively mitigate the burn‐in phenomenon and offer higher stability [119, 120]. Indeed, OS‐based photoelectrodes utilizing NFAs have shown stable operation without the initial rapid performance decline. Yao et al. reported a photocathode where the acceptor material was changed from fullerene to perylene diimide‐based NFA (PDI‐V), as shown in Figure 2A [121]. The active layer with NFAs exhibited significantly reduced photogenerated charge accumulation, enabling stable photoelectrode operation for over 12 h. Similarly, Seo et al. developed a photocathode using EH‐IDTBR, a representative NFA material, which demonstrated world‐class stability for OS‐based photoelectrodes [22]. These studies illustrate that effective suppression of initial burn‐in significantly impacts the overall stability of OS‐based photoelectrodes.

#### 3.1.2 Halide Perovskites

Early studies conducted by Shao et al. revealed that defects in halide perovskite (HP) materials lead to the formation of high‐density deep level traps [124]. The deep level traps not only cause strong non‐radiative recombination of charge carriers within the material, leading to a loss of charge carriers in solar cells, but also induce ongoing degradation phenomena within the material [125, 126]. Therefore, to produce highly stable HP‐based solar cells and photoelectrodes, it is essential to minimize and suppress defects in HP materials that induce deep level traps through effective passivation methods.

One of the most effective reported techniques is passivation using ionic bonding. This method involves neutralizing specific defect sites with counter ions, and numerous studies have demonstrated that this passivation technique enhances the stability of HP‐based devices. Cationic species used as passivating agents can compensate for anionic defects such as undercoordinated I^–^, anti‐site PbI_3_^–^, and MA^+^ vacancies within HP films. Bi et al. and Abdi‐Jalebi et al. reported that small cations like Na^+^ and K^+^ effectively passivated defects at grain boundaries in HP films, thereby improving photoluminescence quantum yield (PLQY) [127, 128]. Lee et al. reported using a larger cation, phenethylammonium (PEA^+^), as a passivating agent for (HC(NH_2_)_2_PbI_3_)_0.85_(CH_3_NH_3_PbBr_3_)_0.15_ HP‐based devices [129]. Observations using Kelvin Probe Force Microscopy (KPFM) indicated that while the grain size decreased, the contact potential difference (CPD) revealed that grain boundaries were passivated with PEA cations, reducing photogenerated charge recombination [130]. This resulted in increased PL intensity, carrier lifetime, and device open‐circuit voltage (*V*_OC_), effectively reducing degradation phenomena.

Additionally, Krishna et al. applied the short‐chain cationic surfactant tetrapropylammonium (TPA^+^) to MAPbI_3_ HP. TPA^+^ ions strongly interacted with the perovskite crystal surface, stabilizing uncoordinated halide ions and passivating surface defects, as shown in Figure 2B [122]. X‐ray diffraction (XRD) analysis of HP films with TPA^+^ revealed that TPA^+^ ions interacted with the lead iodide framework, reducing PbI_2_ excess and forming a new TPAPb_4_I_9_ phase, as shown in Figure 2C. Although TPA^+^ did not integrate into the perovskite lattice, it modified the cell volume and formed TPA^+^‐rich domains at grain boundaries [131]. This passivated device showed significantly enhanced stability against humidity, temperature, and light stress without external encapsulation, as shown in Figure 2D.

Anionic species can also be used as passivating agents, though their application is less varied. Commonly used methods involve introducing chlorides and iodides, such as PbCl_2_, NH_4_Cl, MACl, FACl, KI, and MAI, to compensate for complementary charge defects like Pb^2+^ and halide vacancies, thus serving passivation roles. However, due to the selective nature of these cationic and anionic passivation methods, recent research has focused on using zwitterions, which possess both positive and negative functional groups. Kim et al. used the l‐proline (Pro) zwitterion to passivate MAPbI_3_ HP films, creating highly efficient and stable HP solar cells and photoelectrodes [123]. Pro, with its positively charged ammonium cation and negatively charged carboxylate anion, effectively neutralized two types of ionic defects in HP films, improving photoluminescence and photovoltage, as shown in Figure 2E. The resulting photoelectrode demonstrated an operational stability exceeding 50 h, one of the best reported at that time, as shown in Figure 2F. This study confirmed that ionic bonding‐based passivation positively impacts the operational stability of HP‐based devices when used as photoelectrodes.

Interestingly, in addition to ionic bonding‐based passivation, various molecular passivation agents have shown improved operational stability and performance in HP films when larger molecules are used [132, 133, 134]. This phenomenon occurs because the intercalation of larger molecules disrupts the original 3D structure of HP films, forming 2D or quasi‐2D perovskite structures on the surface and within the film [135, 136, 137]. The 2D perovskite structures not only compensate for defects in HP films but also have a wider bandgap compared to the 3D structures, forming type‐I heterojunctions at the interfaces [138]. These type‐I heterojunctions create electronic barriers that impede charge transport between perovskite grains and between the perovskite and charge transport layers [139, 140]. Consequently, charges reaching the wide‐bandgap wrapping layer are reflected back into the bulk, effectively reducing recombination [141]. Recently, intentional mixing of 2D perovskites with 3D perovskites or fabricating layered structures based on this principle has been actively researched [142, 143, 144, 145].

### 3.2 CTL/Active Layer Interfaces

The HTL must extract holes from the active layer while blocking electrons to prevent recombination of holes and electrons. These two characteristics are intrinsically related to the energy band structure of the HTL. A highest occupied molecular orbital (HOMO) level sufficiently close to the valence band of the active layer allows efficient hole transport. Additionally, the hole mobility in the HTL must be relatively high to minimize charge loss during transport to the contact electrode. However, defects at the HTL/active layer interface paradoxically act as recombination sites for electrons and holes, limiting the hole extraction capability of the HTL. Furthermore, chemical interactions between the HTL and the active layer are another major cause of degradation at the interface. Specifically, ions from the active layer can diffuse into the HTL, or materials from the HTL can penetrate the active layer [146]. These chemical reactions result in structural changes at the interface, causing performance degradation over time. In summary, by preventing defects and anomalies at the HTL/active layer interface, ensuring appropriate energy band alignment and high hole mobility, and enabling smooth hole extraction and electron blocking, the stability of OS and HP‐based devices can be enhanced.

**FIGURE 3:**
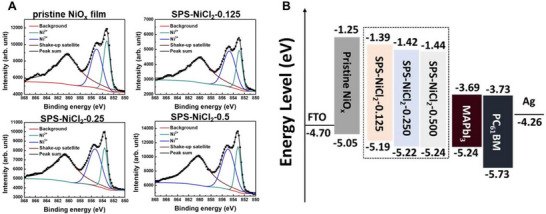
(A) X‐ray photoelectron spectra (XPS) of pristine NiO_x_ film and SPS‐NiCl_2_ treated films. (B) Band alignment of perovskite device with NiO_x_ and NiO_x_ with SPS‐NiCl_2_. Reproduced with permission [147]. Copyright 2021, Elsevier.

Hsiao et al. introduced a sequential passivation strategy with NiCl_2_ (SPS‐NiCl_2_ treatment) on NiO_x_, a representative material for oxide‐based HTL, to induce an appropriate gradient in the valence band maximum (VBM) [147]. The SPS‐NiCl_2_ treated NiO_x_ film had a high Ni^3+^ ratio and low defect density, exhibiting a depth‐dependent VBM gradient, as shown in Figure 3. This VBM gradient could be adjusted to achieve proper energy band alignment with the HP film, providing excellent photovoltaic performance and long‐term stability for the device. This study demonstrates that aligning the surface elements of the HTL properly can tune the energy alignment (VBM gradient) to match the active layer, offering an effective charge transport path and significantly contributing to improved stability. Ding et al. addressed the thermal instability occurring at the MoO_x_ and OS active layer interface [148]. They identified that the abnormal oxidation‐reduction reactions centered on Cl^−^ ions of OS material, when directly in contact with MoO_x_, were the main cause of degradation and inserted a C_70_ intermediate layer. The C_70_ prevented direct contact between MoO_x_ and OS, avoiding ion loss and showing remarkable improvement in operational stability.

**FIGURE 4:**
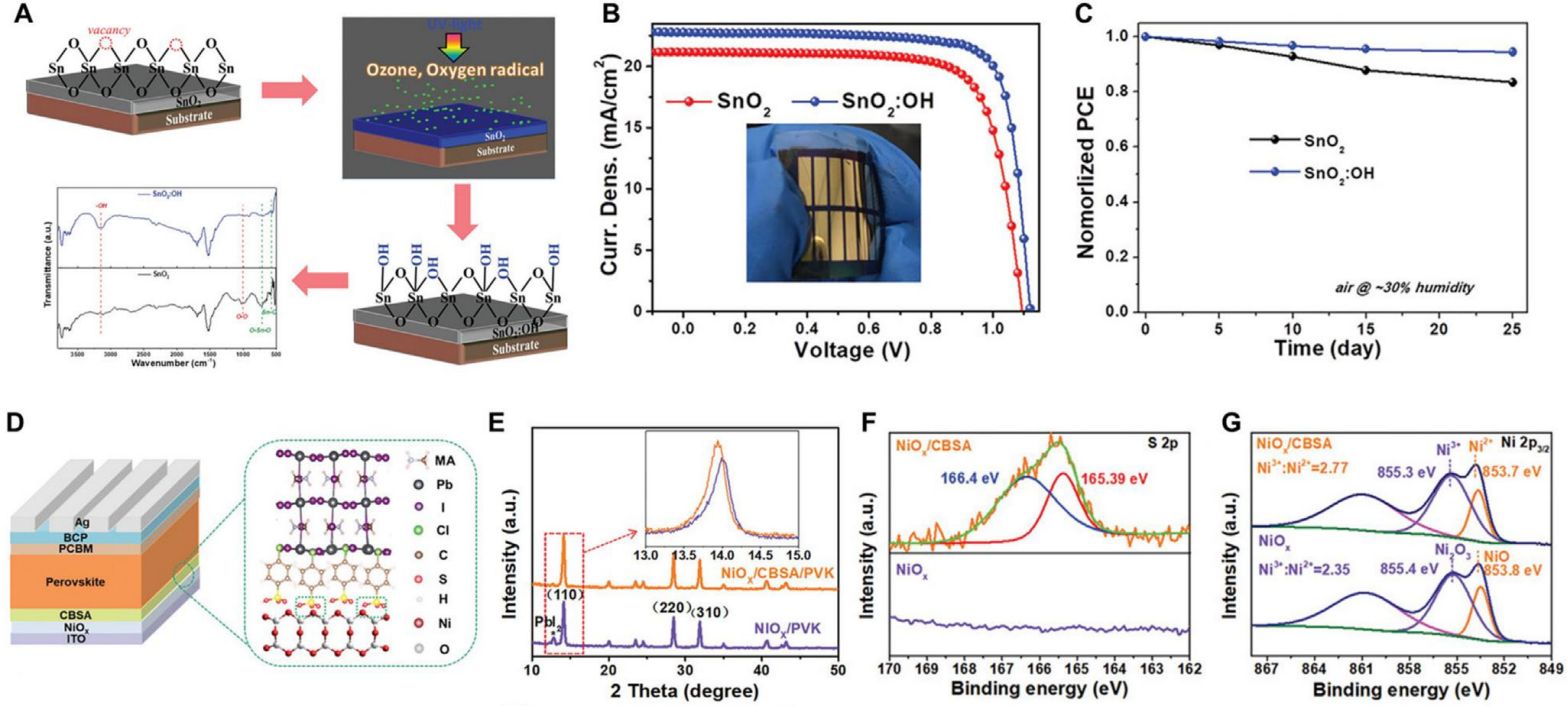
(A–C) Oxygen radical treatment process of SnO_2_ films, and the solar cell performances of HP‐based devices with SnO_2_ and oxygen radical treated SnO_2_ film (SnO_2_:OH). Reproduced with permission [152]. Copyright 2023, Wiley‐VCH. (D–G) Schematic image of PVSCs based on NiO_x_/CBSA, and XRD and XPS spectra change corresponding on CBSA treatment. Reproduced with permission [153]. Copyright 2022, Wiley‐VCH.

Degradation at the ETL/active layer interface shows phenomena similar to those at the HTL interface. However, a notable point is that most of the primary materials used in ETL are inorganic metal oxides. This is because the organic ETL materials developed so far do not show satisfactory electron mobility compared to metal oxides, and their energy level alignment is not ideal. These metal oxides inevitably form oxygen vacancies on the surface, which strongly attract electrons, creating fatal trap states and causing severe charge accumulation at the ETL/active layer interface. [149, 150] Kim et al. performed chemical modification using 1,2‐ethanedithiol (EDT) to prevent degradation caused by oxygen defects in ZnO ETL [151]. As a result, the suppression of interface defects significantly improved charge collection properties, resulting in increased PCE, and maintained 90% of the initial performance over 1200 h, showing much higher stability compared to ZnO ETL with oxygen defects. Yang et al. developed an ETL by modifying SnO_2_ thin film using oxygen radicals to reduce oxygen vacancies at the interface [152]. Oxygen radical passivation formed an SnO_2_ surface, which additionally formed hydrogen bonds with HP, improving charge transfer and moisture resistance at the interface (Figure 4A). Thus, long‐term operational stability was ensured, and it could be uniformly applied to large‐area modules, achieving an efficiency of 18.71%, as shown in Figure 4B,C. Passivating the surface oxygen defects of metal oxide ETLs has been shown to have a remarkably positive impact on device stability.

When using metal oxide CTLs, it is important to note that lattice mismatches can occur at the interface with HP. Lattice mismatches between CTL and HP can create dangling bonds, which naturally lead to deep‐level traps and induce additional charge recombination [154, 155, 156, 157, 158, 159]. Moreover, lattice mismatches increase ion migration and reduce carrier mobility [160, 161, 162]. These issues can be effectively addressed by applying buffer layers. Various materials such as 2D perovskites [163, 164], metal halides [165, 166], and MXene [167, 168] have been used as buffer layers to achieve high‐efficiency and stable HP‐based devices. Recently, composite technologies have been reported that not only mitigate lattice mismatches but also passivate additional interface bonds. Zhang et al. inserted p‐chlorobenzenesulfonic acid (CBSA) self‐assembled small‐molecule (SASM) at the NiO_x_ HTL/HP interface to provide dual passivation by alleviating lattice mismatches and compensating for interface defects [153]. The chlorine (─Cl) functional group of SASM bonded with iodine defects of HP, and the sulfonic acid (─SO_3_H) groups bonded with oxygen vacancies of HTL, thereby suppressing lattice mismatches with an intermediate layer and compensating for surface defects, which were confirmed by XRD and XPS spectra (Figure 4E–G). The HP‐based solar cells with dual passivation showed the highest PCE values at the time and exhibited ideal charge behavior over a long period.

Passivating intrinsic degradation is fundamental to the long‐term operation of OS and HP‐based photoelectrodes. However, intrinsic degradation in OS and HP solar cells and photoelectrodes occurs simultaneously at various locations. Besides the areas described above, degradation can also occur within the CTL [169, 170], at the CTL/electrode interface [171, 172], and within the electrode [173]. Although appropriate passivation methods have been reported to compensate for these issues, further research is needed on passivation materials that can perform multiple functions simultaneously for effective passivation.

## 4 Extrinsic Degradation Mechanisms and Passivation Methods

**FIGURE 5:**
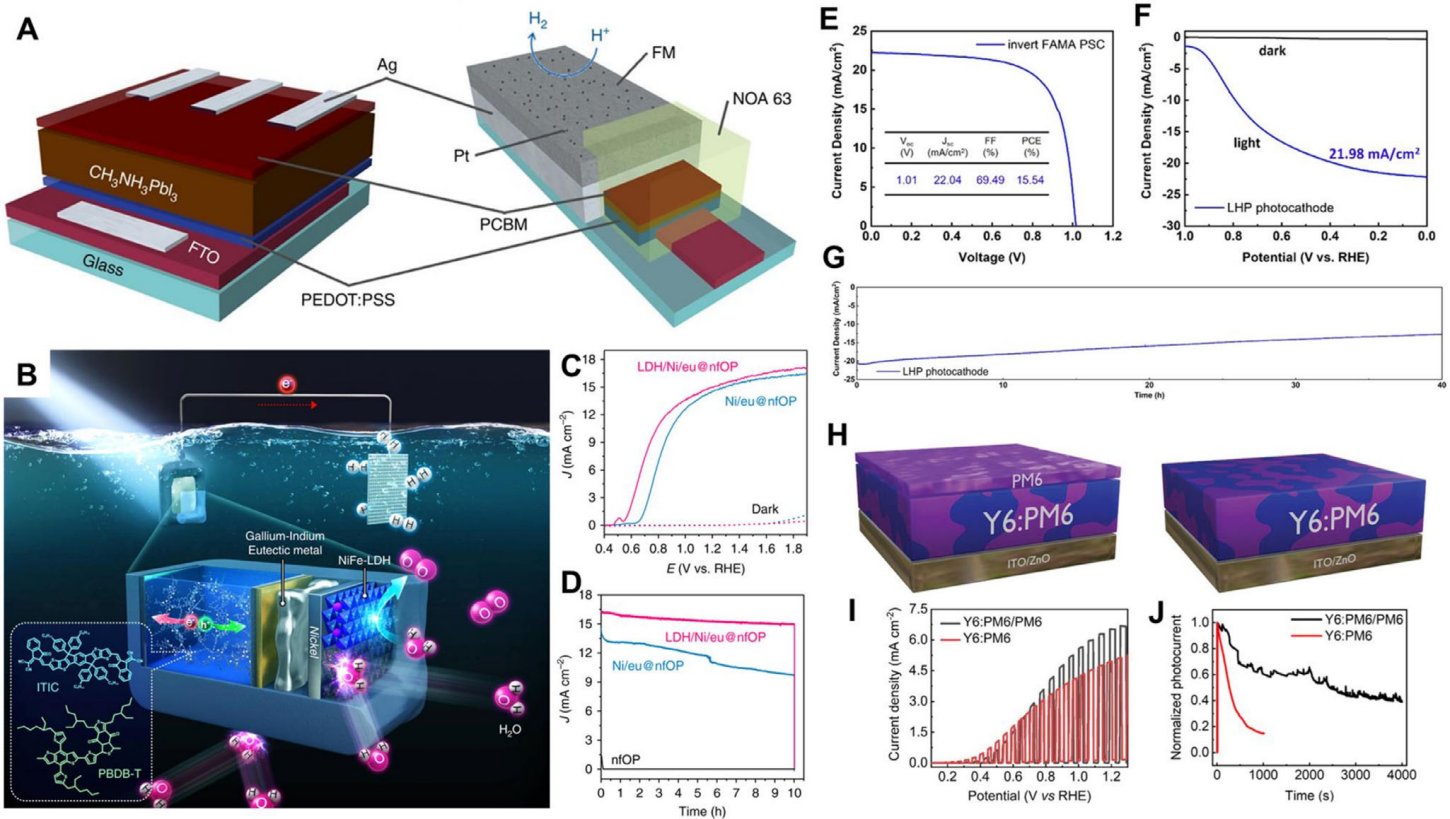
(A) Schematic images of the MAPbI_3_ HP solar cell and metal‐encapsulated HP‐based photocathode. Reproduced with permission [33]. Copyright 2016, Springer Nature. (B–D) Schematic image and the PEC performances of metal‐foil encapsulated OS‐based photoanode with EGaIn adhesion layer. Reproduced with permission [178]. Copyright 2020, Springer Nature. (E–G) The PEC performances of metal‐foil encapsulated HP‐based photocathode with carbon powder adhesive layer. Reproduced with permission [179]. Copyright 2023, Wiley‐VCH. (H–J) Schematic images of polymer overlayer encapsulated OS‐based photoanode, and their PEC performances. Reproduced with permission [180]. Copyright 2023, Wiley‐VCH.

Initial OS and HP‐based photoelectrodes exhibited poor stability, failing within an hour, even with passivation against intrinsic degradation [174, 175, 176, 177]. These photoelectrodes were created by applying catalysts suitable for PEC reactions on the top electrodes of traditional OS and HP‐based solar cell structures. However, the combination of the thin top electrode and catalyst could not prevent electrolyte penetration, leading to extrinsic degradation that caused the structure to dissolve and collapse completely. Consequently, encapsulation methods that could completely block external electrolytes were developed. The first reported technique was metal encapsulation by Crespo‐Quesada et al. [33]. They encapsulated CH_3_NH_3_PbI_3_‐based HP solar cells with InBiSn alloy, known as Field's metal, and a Pt catalyst (Figure 5A). The InBiSn alloy protected the HP structure from the electrolyte and transferred photogenerated electrons to the catalyst, allowing the HP‐based solar cell to operate stably for about 3 h. However, the economic feasibility was unsatisfactory due to the expensive metal materials and noble metal catalysts required for encapsulation. Therefore, researchers began exploring cheaper and more effective encapsulation methods, setting the challenging goal of applying non‐noble metal‐based catalysts to the top of OS and HP‐based solar cells stably.

The proposed solution was to develop an encapsulation part that prevents electrolyte penetration, separate from the OS and HP solar cell device, and use a highly conductive contact material as the adhesion layer. This method adds high flexibility in introducing and synthesizing catalysts in the encapsulation part and can achieve high efficiency with the proper adhesion layer. Thus, the metal‐foil encapsulation technique was developed. Unlike conventional metal encapsulation, it used metal‐foils with non‐noble metal catalysts to fundamentally block electrolyte penetration. Yu et al. reported a photoanode with metal‐foil encapsulation technology applied to a PBDB‐T:ITIC OS‐based solar cell, demonstrating long‐term operation in an electrolyte environment, as shown in Figure 5B [178]. They used Ni foil with the high‐efficiency non‐noble metal catalyst NiFe‐LDH as the electrolyte barrier layer and Ga‐In eutectic alloy (EGaIn) in the gap between the OS‐based solar cell and the metal‐foil. This photoanode achieved a current density performance of 15.1 mA cm^−2^ at 1.23 V versus reversible hydrogen electrode (*V*_RHE_), which is challenging for conventional oxide‐based PEC photoelectrodes, and showed an excellent onset voltage around 0.4 V_RHE_. Remarkably, it maintained 90% of its initial current density for over 10 h at 1.3 V_RHE_. EGaIn, as the adhesion layer, not only exhibited high conductivity but also, being a liquid metal, effectively filled the gap between the top electrode of the OS and HP solar cells and the encapsulation layer. The outstanding performance of metal‐foil encapsulation using EGaIn and other fusible metals has also been reported in other studies [181, 182, 183]. Seo et al. applied Ti‐foil and EGaIn as the encapsulation layer and adhesion layer, respectively, to PTB7‐Th:EH‐IDTBR OS‐based solar cells, creating a photocathode that maintained over 90% of its initial current density for 30 h under 0 *V*_RHE_ conditions, showcasing the world's best operational stability [22]. Choi et al. used a fusible metal adhesion layer with a Ni foil deposited with Fe‐doped Ni_3_S_2_ oxygen evolution reaction catalyst on HP‐based solar cells to create a photoanode [184]. This photoanode achieved an applied bias photon‐to‐current efficiency (ABPE) of 12.79%, the highest among single active layer‐based photoanodes, and maintained about 90% of its initial current density for 12 h at 1.23 *V*_RHE_.

Additionally, EGaIn proved to be much more stable and efficient as an adhesion layer than Ag paste used in organic solvent‐based applications, particularly under conditions where photovoltage is present [123]. However, EGaIn adhesion layers have the drawback of inducing reactions and corrosion with the top electrode of the solar cell to maintain the alloy properties. Over time, the top electrode corrodes, and when the corrosion completes, the contact between the active layer and EGaIn can lead to device failure. Also, the high cost limits its practical application. Therefore, various materials applicable to the adhesion layer have emerged to ensure the ideal encapsulation performance of metal‐foil encapsulation.

Recently, carbon‐based materials such as graphite epoxy [185, 186], carbon powder [187], carbon paste [188], and commercial adhesive [189] have gained attention due to their low cost and excellent conductivity, which does not significantly degrade the photoelectrode's performance. These materials also react less with the top electrode and encapsulation, significantly contributing to improving the cost‐efficiency and operational stability of OS and HP‐based photoelectrodes. Rhee et al. reported HP‐based photoanodes and photoanodes using carbon powder as the adhesive layer [179]. The photocurrent density under zero‐bias conditions of the completed photoelectrode showed a difference of less than 0.1 mA cm^−2^ from the short‐circuit current density of the solar cell, indicating that the relatively low conductivity of the carbon‐based adhesive layer hardly affects the overall device performance, as shown in Figure 5E,F. Additionally, the low reactivity of the adhesive layer with the top electrode of the HP solar cell demonstrated excellent operational stability for over 40 h (Figure 5G).

Recent studies have also aimed to replace the encapsulation layer with low‐cost materials, considering the practical application of robust encapsulation methods. Examples include carbon‐based materials such as graphite sheets [188, 190], carbon paper [189], and carbon plates [191]. An interesting study reported by Jiang et al. used FTO substrates. They connected one side of the FTO substrate to the top electrode of the HP solar cell with Ag paste and exposed the other side with a catalyst [192]. This study demonstrated that easily manufactured and cost‐effective conductive oxides are very promising as encapsulation layers.

Finally, a novel study on the encapsulation of OS‐based photoelectrodes has been conducted recently. Lee et al. applied a polymer overlayer with high durability in electrolytes directly onto the top of the active layer as an encapsulation layer, as shown in Figure 5H [180]. It not only extended the short photoexcited charge lifetimes of OS‐based photoelectrodes but also resolved the operational stability issues of organic materials within electrolytes, previously considered a limitation. Their work not only extended the short photoexcited charge lifetime of OS‐based photoelectrodes, but also addressed the issue of operational stability of organic materials in electrolytes, which was previously considered a limitation. The encapsulated photoanodes exhibited higher photocurrent densities than samples without the polymer overlayer and showed a four‐fold improvement in stability, as shown in Figure 5I,J. This study significantly impacted the ongoing encapsulation research, indicating that organic‐based overlayers could operate more stably from a charge perspective than other inorganic or metal‐based overlayers. Thus, designing and applying new molecules more suitable for PEC and electrolyte conditions could further enhance the performance of OS and HP‐based photoelectrodes.

## 5 PEC Applications of OS and HP Based Photoelectrodes

Applying appropriate passivation and encapsulation has successfully delayed intrinsic and extrinsic degradation in OS and HP‐based photoelectrodes. These electrodes, leveraging their excellent inherent photoelectric properties, can contribute significantly to various cases in the PEC field. The high photovoltage and photocurrent density they exhibit provide a different level of PEC reaction efficiency compared to single light‐absorbing layer photoelectrodes made from traditional oxides and semiconductor materials. This chapter will explore the practical applications of OS and HP‐based photoelectrodes in the PEC field and discuss the achievements in each area.

### 5.1 PEC Water Splitting

PEC water splitting is the most extensively studied topic in the PEC field. In a PEC water splitting system, the cathode is responsible for the hydrogen evolution reaction (HER), and the anode handles the oxygen evolution reaction (OER) [193, 194]. The ultimate goal of this system is to convert solar energy into hydrogen (H_2_), a clean and storable energy source, to build a sustainable society. The HER and OER occur at a 2:1 ratio, with the HER consuming 2 electrons and the OER consuming 4 holes. Therefore, both HER and OER must proceed smoothly to develop a high‐efficiency system [195, 196, 197]. The high absorption coefficients and excellent solar‐to‐power conversion efficiencies of OS and HP‐based photoelectrodes have garnered significant attention in the PEC water splitting field. Consequently, these materials have been actively applied in both photocathode and photoanode areas.

**FIGURE 6:**
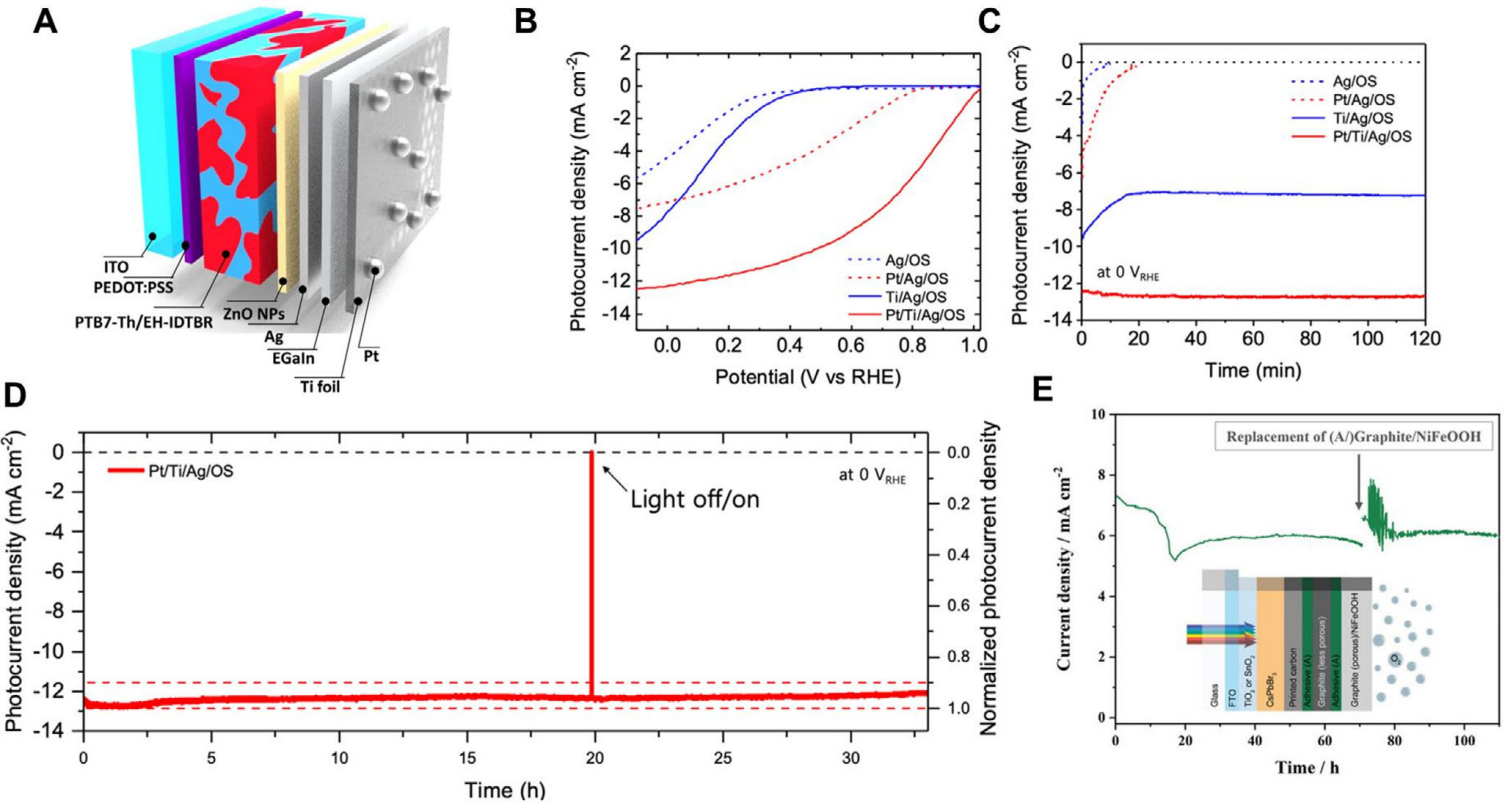
(A–D) Schematic images of the metal‐encapsulated OS‐based photocathode with EGaIn adhesive layer, and the PEC performances of various encapsulated OS‐based photocathodes. Reproduced with permission [22]. Copyright 2022, Royal Society of Chemistry. (E) Long‐term operational stability of NiFeOOH/graphite sheet encapsulated HP‐based photoanode. Reproduced with permission [190]. Copyright 2023, Wiley‐VCH.

In the field of photocathodes, OS and HP‐based photoelectrodes have achieved very high photocurrent densities, on the order of tens of mA cm^−2^, for the HER at 0 *V*_RHE_. Seo et al. developed an OS‐based photocathode with metal‐foil encapsulation, achieving a remarkable photocurrent density of 12.3 mA cm^−2^ at 0 *V*_RHE_, and nearly 100% Faradaic efficiency for HER, indicating that the photoexcited charges were not consumed in degradation reactions within the material but participated in the HER reaction, as shown in Figure 6A–C [22]. The developed photocathode showed less than 10% performance degradation after more than 30 h in 0.5 m H_2_SO_4_ (pH 1), demonstrating high stability, as shown in Figure 6D. The half‐cell solar‐to‐hydrogen (HC‐STH) efficiency reached 5.3%. HP‐based photocathodes, which absorb a wider range of wavelengths than OS, have been reported to achieve up to 20 mA cm^−2^ [123, 183, 198].

Photoanodes require more meticulous approaches due to the OER needing twice as many charges as the HER, resulting in slower reaction kinetics [199]. The slow kinetics of OER impose additional stresses like charge accumulation or recombination on the photoanode [200, 201]. To address this, both materials and catalysts need to be enhanced. Yu et al. used a high‐performance NiFe‐LDH OER catalyst on an OS‐based photoanode with metal‐foil encapsulation and an EGaIn adhesion layer [178]. When only Ni foil without a catalyst layer was used, the stability of the photoanode decreased. Additionally, considering that stability improved in an electrolyte containing a hole scavenger, which requires fewer holes than the OER, it can be understood that in multi‐charge PEC reactions like the OER, the performance of the catalyst significantly impacts the stability of the photoelectrode, in addition to material passivation and encapsulation. Daboczi et al. reported that the highly stable HP‐based photoanodes with graphite sheet encapsulation [190]. Phase engineering of the CsPbBr_3_ HP film reduced intrinsic degradation, and graphite sheets with NiFeOOH catalysts reduced charge accumulation, resulting in 100 h of device failure‐free operation, as shown in Figure 6E. This study is a successful application of maximizing photoelectrode operation stability by proper passivation and encapsulation and the introduction of highly efficient HER and OER catalysts.

### 5.2 PEC CO_2_ Reduction Reaction

PEC carbon dioxide reduction reaction (CO_2_RR) aims to reduce atmospheric CO_2_ levels, heightened by fossil fuel usage, by mimicking the carbon cycle process of photosynthesis. Known as artificial photosynthesis, this system focuses on converting excess CO_2_ into valuable chemicals such as formic acid (HCOOH), carbon monoxide (CO), methane, methanol (CH_3_OH), ethanol (C_2_H_5_OH), and ethylene (C_2_H_4_). Key challenges in PEC CO_2_RR include addressing the thermodynamic overpotential required for CO_2_RR and maintaining selectivity against competing HER [202, 203].

**FIGURE 7:**
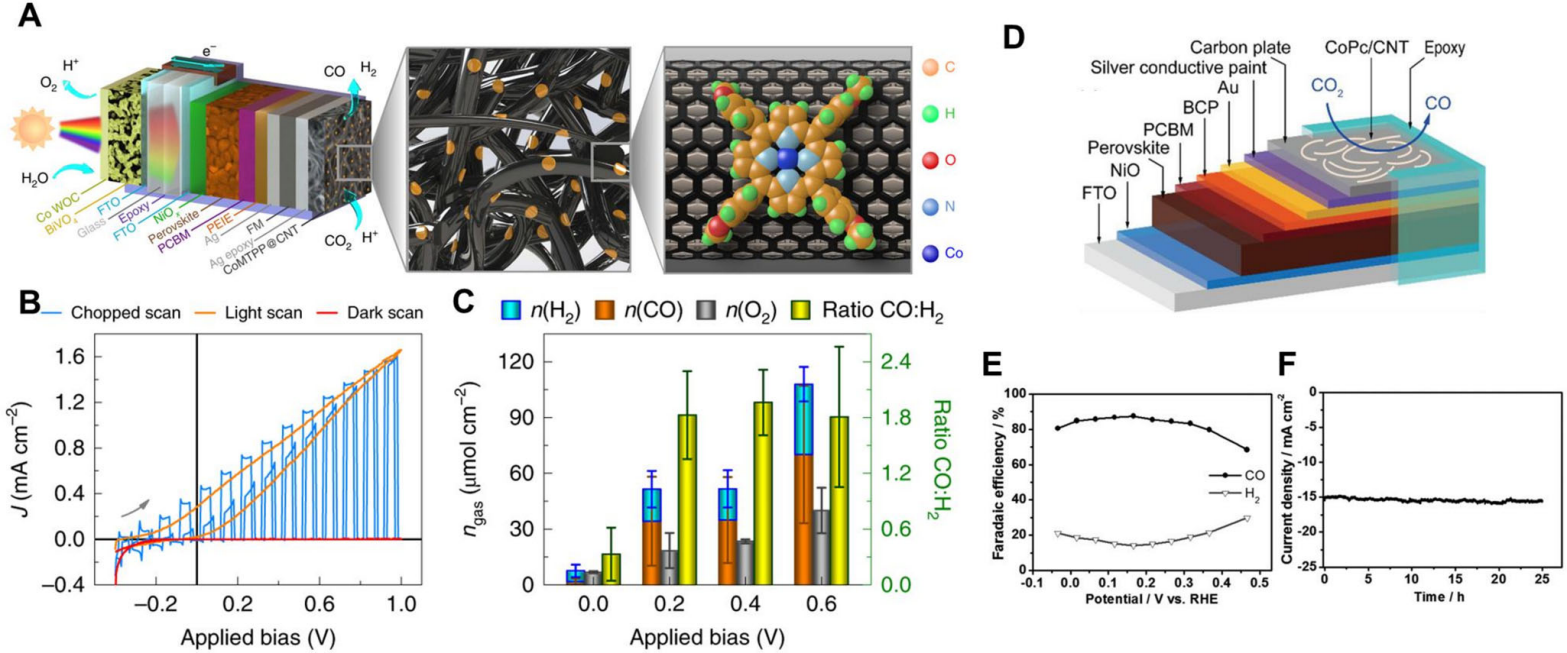
(A–C) Schematic images of the HP‐BiVO_4_ tandem structure, and the PEC CO_2_RR performances of HP tandem‐based photocathodes. Reproduced with permission [205]. Copyright 2020, Springer Nature. (D–E) Schematic image of CoPc/carbon plate encapsulated HP‐based photocathodes, and their PEC CO_2_RR performances. Reproduced with permission [191]. Copyright 2020, Wiley‐VCH.

Chen et al. reported that CH_3_NH_3_PbI_3_ photoelectrodes encapsulated with In_0.4_Bi_0.6_ alloy generated sufficient photovoltage to reduce CO_2_ to formic acid at −0.6 V_RHE_, demonstrating the ability of OS and HP‐based photoelectrodes to overcome the overpotential for CO_2_RR [204]. It was the first result to prove that the high photovoltage and effective photoexcited charge characteristics of OS and HP‐based photoelectrodes significantly help in addressing the overpotential required for CO_2_RR. Andrei et al. further developed more efficient HP‐based photoelectrodes, achieving PEC CO_2_RR at 0 *V*_RHE_ as shown in Figure 7A,B [205]. Also, they fabricated a tandem cell with BiVO_4_ layer to realize bias‐free PEC system, and achieved 1.96 ± 0.35 maximum produced CO:H_2_ ratio, as shown in Figure 7C. However, the efficiencies achieved were limited due to the inadequate reaction activity of the catalysts and unsatisfactory performance of the photoelectrodes. Zhang et al. developed a Cs_0.15_FA_0.85_Pb(I_0.9_Br_0.1_)_3_ photocathode encapsulated with a carbon plate [191]. Carbon plate served as an excellent support for a high‐performance molecular CO_2_RR catalyst, cobalt phthalocyanine (CoPc), enhancing the photocathode's stability and efficiency, as shown in Figure 7D. The CoPc decorated HP‐based photocathode shows the Faradaic efficiency of CO production was 68.4% at 0.47 *V*_RHE_ and increased to 87.5% at 0.17 *V*_RHE_ with long‐term operational stability for 25 h, as shown in Figure 7D–F. They demonstrated the highest efficiency reported for CO_2_ reduction photoelectrodes at the time, emphasizing the importance of selecting appropriate encapsulation layers and catalysts for high‐efficiency CO_2_RR. However, a more in‐depth approach to producing a wider variety of high‐value carbon compounds is needed.

### 5.3 PEC Ammonia Production

Ammonia (NH_3_) is crucial for various industrial applications, particularly as a key component of fertilizers in agriculture [206, 207, 208]. However, the traditional Haber–Bosch process for NH_3_ production is energy‐intensive and heavily reliant on fossil fuels, contributing significantly to greenhouse gas emissions and environmental degradation [209, 210, 211]. PEC NH_3_ synthesis aims to produce NH_3_ in a more sustainable and energy‐efficient manner using solar energy, potentially reducing greenhouse gas emissions and overall energy consumption [212, 213, 214]. To produce NH_3_ via PEC systems, the electrolyte must contain a nitrogen source. Initially, nitrogen gas was purged into the electrolyte, but the low solubility of nitrogen gas and the difficulty of reducing its triple bond limited the yield and Faradaic efficiency [212, 215]. Recent efforts have shifted to using nitrate (NO_3_^−^) as the nitrogen source, expecting higher efficiency PEC NH_3_ production. However, existing photoelectrodes still show limited results, achieving only a few mA cm^−2^ [216, 217].

**FIGURE 8:**
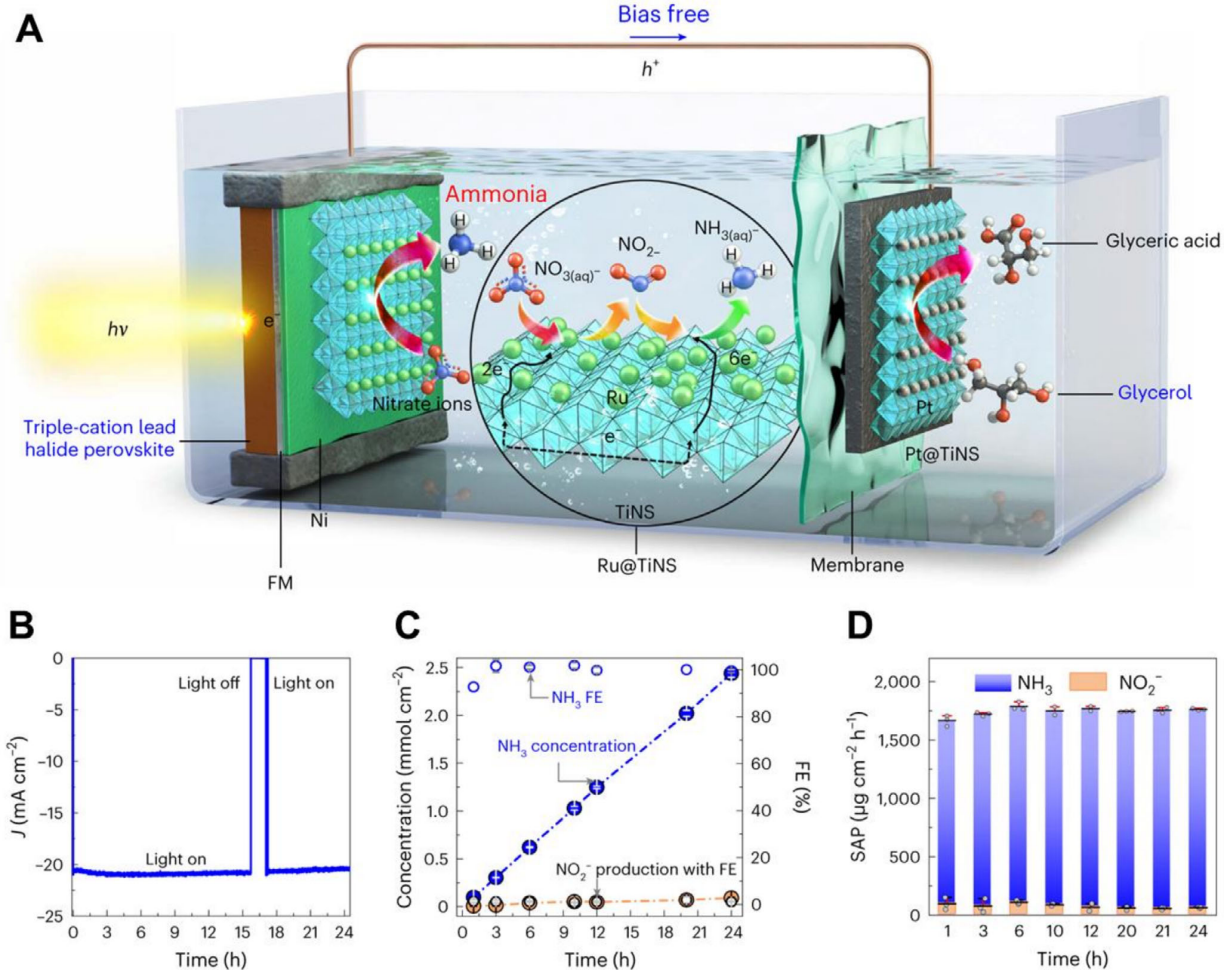
Schematic images of the PEC NH_3_ production based on HP‐photocathode with glycerol valorization system, and their PEC performances. Reproduced with permission [218]. Copyright 2024, Springer Nature.

Tayyebi et al. used Cs_0.05_(FA_0.83_MA_0.17_)_0.95_Pb(Br_0.17_I_0.83_)_3_ HP‐based photoelectrodes encapsulated with Ni foil and Field's metal to produce NH_3_ from NO_3_^−^, as shown in Figure 7 [218]. They modified the anodic oxidation reaction from OER to glycerol oxidation, reducing the overall system overpotential and achieving bias‐free solar‐driven NH_3_ production with a solar‐to‐ammonia productivity of 1744.9 ± 20.6 µg cm^−2^ h^−1^, the highest reported for PEC NH_3_ production (Figure 8D). This report demonstrates that HP‐based photoelectrodes can significantly impact the yield and efficiency of PEC NH_3_ production. The excellent scalability of OS and HP materials is expected to further enhance the yield of these high‐value‐added materials.

### 5.4 Bias‐Free Systems

The ultimate goal of all PEC research areas is to develop a bias‐free PEC system driven solely by photovoltage without an external voltage. Traditional bias‐free PEC systems, constructed from low bandgap oxides like BiVO_4_ and hematite in tandem with Si‐based solar cells [219, 220]. They have shown reasonable photovoltage but insufficient to drive PEC systems, with inherently low photocurrent densities necessitating the tandem approach. However, for achieving high efficiency while minimizing system complexity, this approach is less ideal [221, 222]. The excellent photovoltage and charge generation efficiencies of OS and HP materials significantly contribute to this goal [179]. OS and HP‐based photoelectrodes achieve around 1.0 V of onset potential shift with a single light‐absorbing layer and higher photocurrent densities than oxide photoelectrodes. With various passivation and encapsulation techniques, these materials can operate stably for extended periods. Despite this, achieving bias‐free PEC operation with a single photoelectrode remains challenging, leading to the adoption of dual photoelectrode systems using OS and HP materials.

**FIGURE 9:**
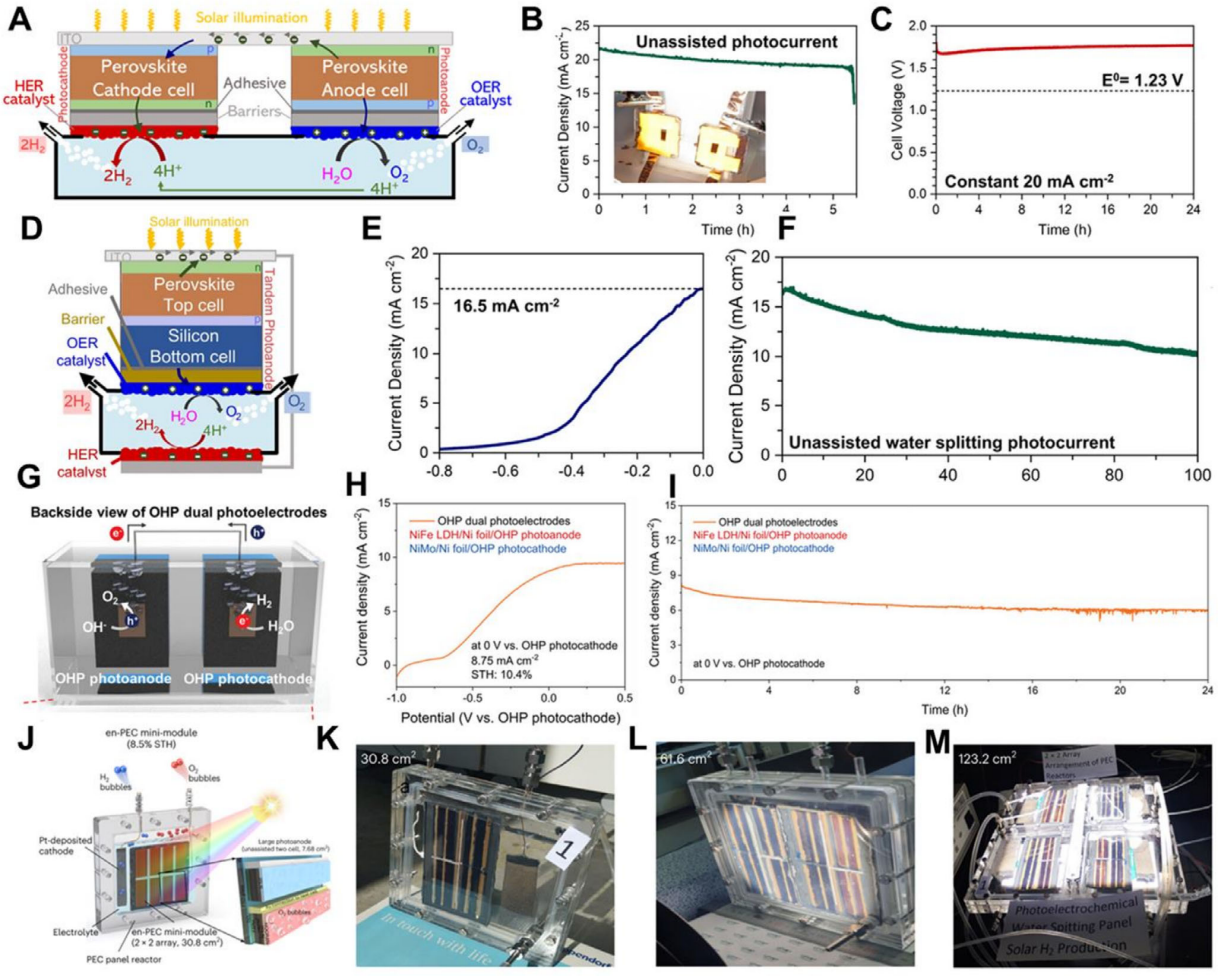
(A–C) Schematic images of the HP based dual‐photoelectrodes system for the bias‐free water splitting, and their PEC performances. (D–F) Schematic images of the HP/Si tandem‐based photoelectrode system for the bias‐free water splitting, and their PEC performances. Reproduced with permission [223]. Copyright 2023, Springer Nature. (G–I) Schematic images of the HP based dual‐photoelectrodes system with transition metal catalysts for the bias‐free water splitting, and their PEC performances. Reproduced with permission [186]. Copyright 2023, Wiley‐VCH. (J–M) Schematic images of the HP based‐single module photoelectrode, and the photographs of the multi‐module photoelectrodes for the bias‐free water splitting. Reproduced with permission [225]. Copyright 2024, Springer Nature.

Fehr et al. applied HP photoelectrodes encapsulated with carbon adhesive and graphite sheets to a dual‐photoelectrode system, realizing bias‐free PEC water splitting [223]. Unlike conventional dual photoelectrode systems, superior photoelectric properties of HP materials resulted in a very high photocurrent density of 20 mA cm^−2^ at one photoelectrode and an outstanding STH efficiency of 12.4 ± 0.7%, as shown in Figure 9A–C. Achieving over 10% STH efficiency is considered the minimum for PEC system commercialization [224]. There HP‐based dual‐photoelectrode system was one of the first systems to achieve an STH efficiency of over 10%. When these HP materials were connected in tandem with Si solar cells in a conventional bias‐free system, they achieved an STH efficiency of approximately 20%, an unprecedented efficiency, and demonstrated commendable stability (Figure 9D–F). This study clearly shows the significant performance gap between the excellent photoelectric properties of OS and HP materials and those of oxide‐based photoelectrodes. Choi et al. also reported a bias‐free PEC water splitting system with HP‐based dual photoelectrodes with the transition metal catalysts, as shown in Figure 9G–I [186]. They also demonstrated an STH efficiency of over 10%, with the notable achievement of stable operation for 24 h. The excellent stability was attributed to the appropriate use of 2PACz HTL for passivation within the HP solar cell structure, long‐term stable encapsulation based on carbon paste, and the great performance of catalysts specifically designed for the electrochemical reactions at both the anode and cathode.

Hansora et al. maximized the scalability of HP materials, achieving a significant technological breakthrough with large‐area bias‐free PEC water splitting systems, as shown in Figure 9J–M [225]. The research team implemented a range of bias‐free PEC water splitting systems, from single devices (0.25 cm^2^) to mini modules (30.8 cm^2^) and large‐scale modules (61.6 cm^2^, 123.2 cm^2^). They addressed the overpotential required for water splitting solely with HP single devices and single photoelectrodes by connecting the HP solar cells in series and enhanced the current values of the system through parallel connections. As a result, they achieved an STH efficiency of around 10% across all sizes, demonstrating the remarkable commercialization potential of the bias‐free PEC system.

## 6 Conclusion and Perspectives

In conclusion, next‐generation PEC systems leveraging OS and HP hold significant promise for efficient solar energy conversion and environmental pollutant upcycling. These materials offer high photovoltage and impressive current densities, significantly enhancing PEC system performance. However, OS and HP‐based photoelectrodes face intrinsic and extrinsic degradation challenges under PEC operating conditions. Intrinsic degradation is driven by photochemical reactions, ion migration, and defects, necessitating various passivation strategies. Extrinsic degradation, caused by electrolyte‐induced corrosion and material dissolution, is addressed through advanced encapsulation techniques. These methods ensure the long‐term stability of OS and HP‐based photoelectrodes, making them viable for diverse PEC applications.

Future research should focus on optimizing the design and synthesis of these materials to enhance their intrinsic stability and performance characteristics. This includes exploring novel organic and perovskite formulations that offer better defect tolerance, increased resistance to photochemical degradation, and improved charge transport properties. Additionally, there is a need to develop more advanced passivation and encapsulation technologies that can provide comprehensive protection against both intrinsic and extrinsic degradation mechanisms. Such advancements could involve multi‐functional coatings, hybrid organic‐inorganic layers, or self‐healing materials that respond dynamically to environmental stressors.

Moreover, scaling up these technologies for practical applications will be crucial for their integration into commercial systems. This involves not only improving material performance but also addressing challenges related to manufacturing, cost, and environmental impact. By advancing these areas, PEC systems based on OS and HP materials could play a pivotal role in sustainable energy solutions, offering efficient pathways for solar‐to‐chemical energy conversion while simultaneously addressing environmental pollutants such as microplastics, organic dyes, and other hazardous waste products.

By focusing on these key areas of research, the field can make significant strides towards the development of more robust, efficient, and sustainable PEC technologies. These innovations will contribute substantially to the global effort to harness renewable energy sources and mitigate environmental pollution, paving the way for a cleaner, more sustainable future.

## Author Contributions

Sanghan Lee supervised the manuscript.

## Conflicts of Interest

The authors declare no conflicts of interest.

## Data Availability

The data that support the findings of this study are available from the corresponding author upon reasonable request.
